# Ag–Cellulose Hybrid Filler for Boosting the Power Output of a Triboelectric Nanogenerator

**DOI:** 10.3390/polym15051295

**Published:** 2023-03-03

**Authors:** Supakit Chenkhunthod, Wimonsiri Yamklang, Walailak Kaeochana, Teerayut Prada, Weeraya Bunriw, Viyada Harnchana

**Affiliations:** 1 Department of Physics, Khon Kaen University, Khon Kaen 40002, Thailand; 2 Institute of Nanomaterials Research and Innovation for Energy (IN-RIE), Khon Kaen University, Khon Kaen 40002, Thailand

**Keywords:** natural rubber, cellulose fibers, Ag nanoparticles, triboelectric nanogenerator

## Abstract

The triboelectric nanogenerator (TENG) is a newly developed energy harvesting technology that can convert mechanical energy into electricity. The TENG has received extensive attention due to its potential applications in diverse fields. In this work, a natural based triboelectric material has been developed from a natural rubber (NR) filled with cellulose fiber (CF) and Ag nanoparticles. Ag nanoparticles are incorporated into cellulose fiber (CF@Ag) and are used as a hybrid filler material for the NR composite to enhance the energy conversion efficiency of TENG. The presence of Ag nanoparticles in the NR-CF@Ag composite is found to improve the electrical power output of the TENG by promoting the electron donating ability of the cellulose filler, resulting in the higher positive tribo-polarity of NR. The NR-CF@Ag TENG shows significant improvement in the output power up to five folds compared to the pristine NR TENG. The findings of this work show a great potential for the development of a biodegradable and sustainable power source by converting mechanical energy into electricity.

## 1. Introduction

With the rapid growth of the Internet of things (IoTs), micro/nano sensors, as well as portable and wearable electronic devices, are extensively developed, requiring massive energy demand. The triboelectric nanogenerator (TENG) is a new energy technology which is promising as an effective power source to sustain these new generation electronics by converting mechanical energy into electricity [1,2]. With a combination of two physical phenomena—contact electrification and electrostatic induction—TENG generates an alternating current electricity with many significant advantages, including high output voltage, cost-effectiveness, a simple fabrication process, and numerous operation modes [3]. This gives TENG the potential for application in diverse fields, such as micro/nano power sources, self-powered sensors, large-scale blue energy, and direct high-voltage power sources.

Biodegradable and biocompatible TENGs have recently received increasing interest due to their potential application in various fields [4]. Numerous natural material-based TENGs were developed, such as those derived from natural wood [5], natural leaves [6], silk [7], cellulose [8,9], lignin [10], and chitosan [11]. Natural rubber (NR) and cellulose are the two natural polymers that have gained increasing attention for the fabrication of biodegradable and biocompatible TENGs [9,12,13,14,15]. NR, or polyisoprene, is a natural polymer with a chemical formula of cis-1, 4-polyisoprene, which is obtained from the *Hevea brasiliensis* tree [16]. NR is used as raw material for the production of many industrial goods due to its good flexibility, high tensile strength, and low cost. The majority of NR products involve their subjection to mechanical force; thus, NR is one of the attractive materials for TENG fabrication, replacing the synthetic polymers such as polytetrafluoroethylene (PTFE) [17], polydimethylsiloxane (PDMS) [18], polyvinylidenefluoride (PVDF) [19], and polyimide (Kapton) [20], which are non-biodegradable and expensive.

According our previous study [21], cellulose fiber (CF) was employed to modify NR, which was found to effectively improve TENG performance due to its electron donating property [22,23], which is preferred for tribopositive materials. Regarding many appealing aspects of cellulose-based materials, including significant bioavailability, good mechanical properties, insolubility in common organic solvents—which is ideal for structural engineering materials—and low cost [24,25,26], it is promising for modifying NR to develop the NR-based TENG with enhanced power output, which is crucial for realizing the potential application of TENGs.

As for improving the energy conversion efficiency of TENG, diverse approaches were proposed to modify triboelectric polymers, including surface/internal structure modification [18,27,28], chemical functionalization [29], and improving charge capacitance of the triboelectric layer by filling it with metal nanoparticles and high dielectric constant materials [18,30,31,32]. For the case of metal nanoparticles filling, Ag nanoparticles were extensively used to enhance the energy conversion efficiency of triboelectric polymers [32,33,34,35,36]. In this work, the hybrid material composed of cellulose fiber and Ag nanoparticles was synthesized and used as a filler for NR triboelectric film. Cellulose fibers were derived from sugarcane leaves, one of the major agricultural wastes from sugarcane farm, which were employed synergistically as a filler for NR and a capping agent for Ag nanoparticles. The Ag nanoparticle content in the composites was optimized. The contribution of CF-Ag hybrid filler was investigated and discussed. The applications of the fabricated NR–CF–Ag TENG as a power source were also demonstrated. Since NR and sugarcane are industrial crops in many countries, the success of this research would contribute to the bio-circular-green economy regarding the development of green and sustainable energy.

## 2. Materials and Methods

### 2.1. Materials and Chemicals

The NR latex was obtained from the Thai Rubber Latex Group Public Co., Ltd. (Chonburi, Thailand). Silver nitrate (AgNO_3_, 99.5%) was purchased from Merck (Mumbai, India). Sodium borohydride (NaBH_4_, 96.0%) was purchased from QReC (Auckland, New Zealand). Sodium hydroxide (NaOH, 97%) was received from KEMAUS (Cherrybrook, Australia). Hydrogen peroxide (H_2_O_2_, 35%) was obtained from ANaPURE (Brightchem Sdn. Bhd., Selangor, Malaysia). Hydrochloric acid solution (HCl, 37%) was obtained from RCI Labscan (Bangkok, Thailand).

### 2.2. Synthesis of Ag-CF Hybrid Material

Cellulose microfibers were synthesized from sugarcane leaves (SL), as described in our previous studies [21,37], which is briefly explained as follows. Dried SL powders were treated in alkaline solution by mixing 30.0 g of SL powder with 10% NaOH at a solid/liquid ratio of 1:20 g/mL at 90 °C for 4 h. The suspension was then filtered and washed several times with DI water to reach a neutral pH. After that, the specimen underwent bleaching treatment using a mixture of 25% H_2_O_2_ and 2% NaOH solutions (at a volume ratio of 4:1) and heated at 90 °C for 3 h. The product was then washed with DI water to obtain a neutral pH. The filtrated product was then hydrolyzed in a 10% HCl at 90 °C for 2 h. Finally, the suspension was washed with DI water to neutral and filtered to achieve a cellulose microfiber (CF) paste.

Ag–CF hybrid material was prepared by mixing 0.24 g CF paste with 2 mL of AgNO_3_ solutions at various concentrations: 1, 2, and 3 mM. The suspension was then added dropwise into 30 mL of chilled NaBH_4_ solution (with the same concentration as the AgNO_3_ solution). A mixture solution with yellow color was obtained, which was stirred for 10 min. The product was then centrifuged to remove excess solution and filtered to obtain Ag-CF composite paste. The specimens were labelled as CF@Ag_1, CF@Ag_2, and CF@Ag_3, for those prepared from the AgNO_3_ at 1, 2, and 3 mM, respectively.

### 2.3. Preparation of NR–CF@Ag Triboelectric Films

The Ag-CF paste was mixed with 20 g NR latex (0.20 wt%), which was magnetically stirred for 10 min. A total of 2 mL of the mixture suspension was cast on a 4 × 4 cm^2^ ITO glass substrate. Three specimens were prepared for each experimental condition. The specimens were then left to dry overnight at room temperature and later kept in an oven at 60 °C for 6 h. The average thickness of the dry films was approximately 2 mm.

### 2.4. Material Characterizations

The morphologies and crystal structure of the CF and NR–CF@Ag composite films were studied using a scanning electron microscope (SEM) (Helios Nanolab, FEI, Waltham, MA, USA) and X-ray diffraction (XRD) analysis (PANalytical EMPYREAN, Malvern, UK). Fourier-transform infrared spectroscopy (FTIR TENSOR27, Bangkok, Thailand) was employed to probe the chemical structure of the composite films. The dielectric properties of the fabricated NR composites were probed using a Keysight E4990 A impedance analyzer (Colorado Springs, CO, USA) at a frequency ranging from 10^2^−10^6^ Hz.

### 2.5. TENG Output Measurement

The electrical outputs of the NR–CF@Ag TENGs were measured under a single electrode mode. A 3 mm thick PTFE sheet was employed as a pair material for TENG performance testing, with the contact area of 4 × 4 cm^2^. The working distance between the surfaces of the PTFE sheet and the NR composite film was 8 cm. The applied impact force for performance testing was generated by a DC motor, which was able to provide the impact force in the range of 1–10 N. The output voltage and current were measured during the impact force of 5 N at a working frequency of 5 Hz using an oscilloscope (Tektronix DPO2002B, Tektronix China Ltd., Shang Hai, China) and a digital ammeter (Keithley DMM6500, Tektronix China Ltd., Shang Hai, China), respectively.

## 3. Results and Discussion

**Figure 1:**
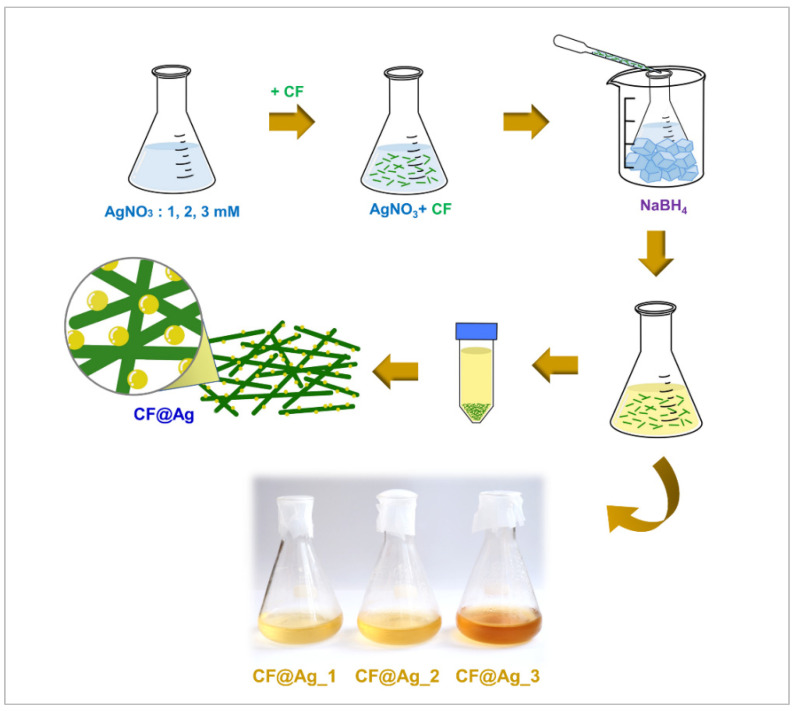
Schematic illustration of the fabrication process of NR–CF@Ag TENG.

**Figure 2:**
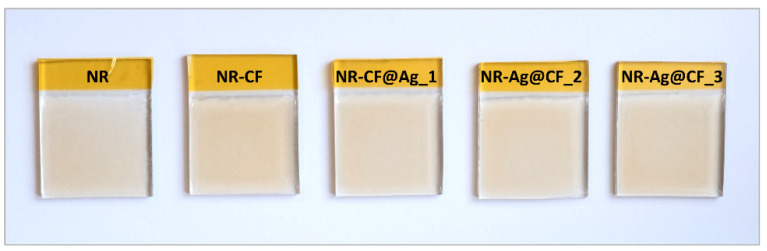
Photographs of all the fabricated TENG including NR, NR–CF, and NR–CF@Ag_1–3.

The fabrication process of the NR–CF@Ag TENG is summarized as illustrated in the schematic in Figure 1. The physical appearances of the prepared CF@Ag suspensions were relatively different in color, where the yellow color became darker, indicating that the number of Ag particles formed increased as the AgNO_3_ concentration increased. However, when the CF@Ag fillers were incorporated into NR, no significant difference was observed among the prepared NR–CF@Ag_1–3 and NR film, as shown in the digital photographs in Figure 2. This was attributed to the fact that the CF@Ag content in all the composite samples was fixed at 0.2 wt% of NR, which was the optimized ratio, as reported in our previous study [21].

**Figure 3:**
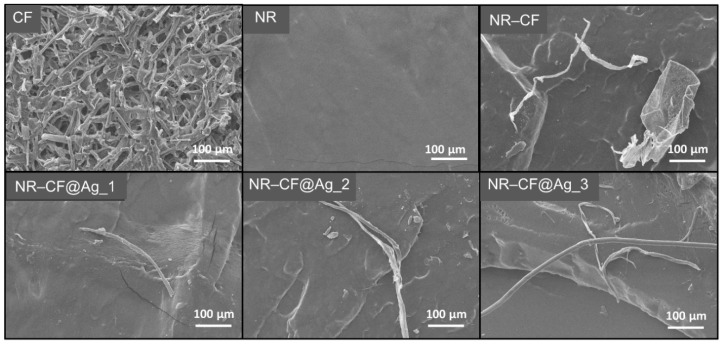
SEM images of the CF and cross-sectional structures of NR, NR–CF, and NR–CF@Ag_1–3 films.

**Figure 4:**
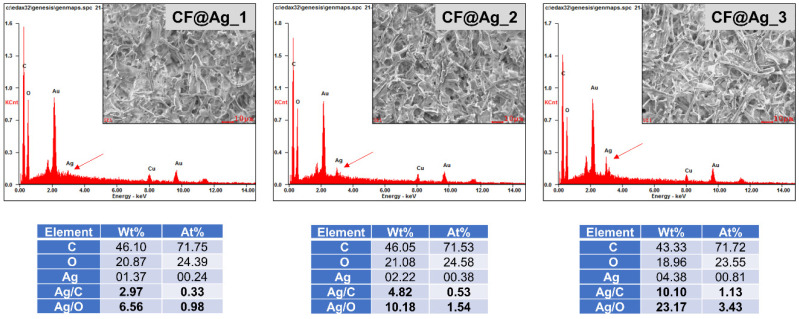
EDX-SEM elemental analysis confirming the presence of Ag nanoparticles in the CF@Ag_1–3 films.

The SEM images in Figure 3 reveal the morphologies of the synthesized CF and the internal (cross-sectioned) structures of the NR, NR–CF, and NR–CF@Ag_1–3 composite films. Cellulose fibers with diameters of a few tens of microns were detected in all the NR–CF composites, including the NR–CF and NR–CF@Ag_1–3 specimens. A small amount of CF was observed in the NR–CF, NR–CF@Ag specimens. This was because the CF content was controlled at 0.20 wt% of NR in all the samples. It was also observed that the fiber sizes were relatively large, which could cause the non-uniform dispersion of the fiber in the NR polymer. Ag nanoparticles were not observed in the SEM images, but the presence of Ag in the CF@Ag specimens was confirmed by the EDX elemental analysis, which was found to increase with increasing AgNO_3_ concentrations, as displayed in Figure 4. The Ag contents in the CF@Ag_1, CF@Ag_2, and CF@Ag_3 were 1.37, 2.22, and 4.38 wt%, respectively.

**Figure 5:**
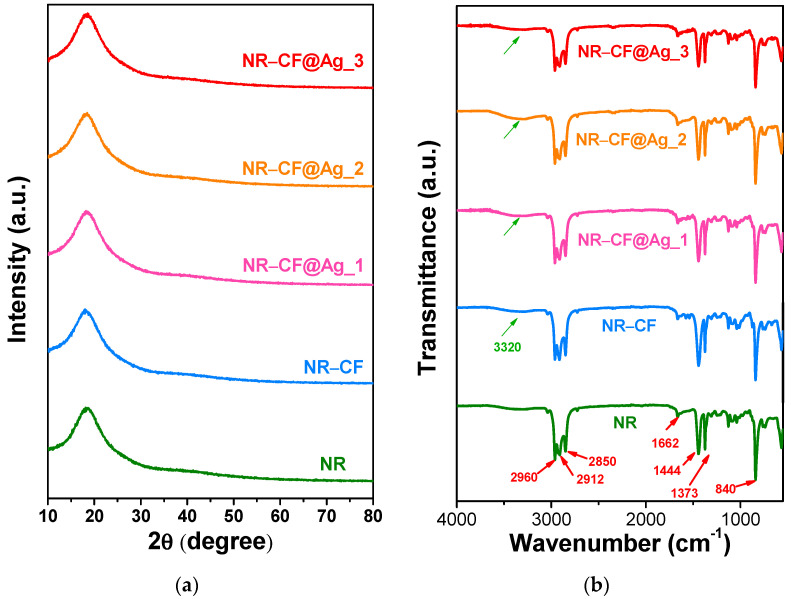
(**a**) XRD spectra and (**b**) FTIR transmittance spectra of NR, NR–CF, and NR–CF@Ag_1–3 composite films.

XRD and FTIR spectra of the NR–CF@Ag_1–3 films compared to those of the pristine NR and NR–CF samples are displayed in Figure 5. XRD patterns of all the specimens indicated that the films had an amorphous structure, with the prominent broad diffraction peak at 2θ~18° (Figure 5a), similar to those of NR reported elsewhere [38,39,40]. No additional filler material peaks were observed, since the amount of CF and Ag nanoparticles were relatively small compared to NR, as discussed above.

The FTIR spectra of all the NR–CF and NR–CF@Ag films were relatively alike and similar to that of pristine NR, as revealed in Figure 5b. The FTIR absorption peaks at 840 and 1662 cm^−1^, which corresponded to the out-of-plane bending vibration of the C–H and C=C stretching of cis-1,4-polyisoprene, respectively [41]. The peaks at 1373 and 1444 cm^−1^ are associated with the CH_2_ deformation, and the multiple peaks at and 2850, 2912, and 2960 cm^−1^ correspond to the asymmetric-symmetric stretching vibration of CH_2_ and C–H in the NR molecule, respectively [42]. The FTIR absorption peak of cellulose was not clearly observed; however, the broad band centered at around 3320 cm^−1^, which corresponded to the stretching vibration of O–H, was found to gain absorption intensity in the NR–CF and NR–CF@Ag films compared to that of the NR sample, indicating the presence of cellulose in the NR–CF-based samples.

**Figure 6:**
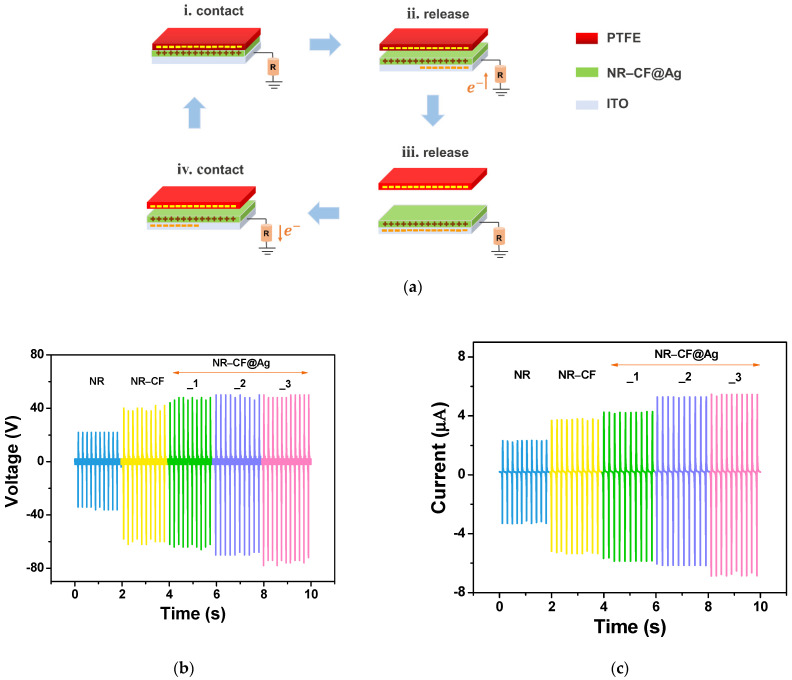
(**a**) The TENG working mechanism in a single electrode mode; (**b**) output voltage and (**c**) current of the NR, NR–CF, and NR–CF@Ag_1–3 TENGs under 5 N impact force at a working frequency of 5 Hz.

Among four basic working modes of TENG—vertical contact-separation mode, single-electrode mode, lateral sliding mode, and freestanding mode—the single electrode mode is easy to fabricate. In this mode, the conductive electrode is attached to one triboelectric material, whereas the contact material is free to move. Thus, the wiring between two triboelectrodes is not required; thus, a single electrode TENG is suitable for scavenging mechanical energy from contact electrification effects from various types of materials [43]. In this work, the NR–CF@Ag TENGs were fabricated and their performances were evaluated under a single electrode mode. The TENG device configuration and working mechanism are illustrated in Figure 6. The NR–CF@Ag films consisted of positive tribomaterial coated on an ITO conductive electrode, which were tested with a PTFE negative tribomaterial. The electricity is generated under the repeated contact-release cycle of PTFE (top) and the NR–CF@Ag (bottom) surfaces. At state i, the electrification effect occurs when two surfaces are in contact, and electrons are transferred from the NR–CF@Ag films to PTFE due to the high electron affinity of PTFE, giving rise to the formation of positive and negative surface charges on the NR–CF@Ag films and PTFE, respectively. When the surfaces are released in states ii-iii, the electrical potential is created, and the electrostatic induction works by inducing free electrons to flow from the ground to the ITO conductive glass to neutralize the potential. At this state, a positive current signal is created. Once the surfaces are back in contact (state iv), the potential is reduced, and free electrons flow back to the ground, generating an electric current in the opposite direction (negative current).

The plots of the output voltage and current signals of all the fabricated TENGs are presented in Figure 6b,c, respectively, and the peak-to-peak output values are given in Table 1. It was observed that the electrical outputs of the TENG were significantly improved after adding CF, which was consistent with our previous study using cellulose nanofibers [21]. The improvement in the electrical output was attributed to the electron donating properties of cellulose due to the presence of oxygen in their hydroxyl functional groups [44,45]. The incorporation of Ag nanoparticles further improved the TENG performance, which increased with increasing Ag content. The highest electrical output voltage and current (peak-to peak) of 128 V and 12.4 µA were obtained from the NR–CF@Ag_1–3 TENGs, which were higher than those of pristine NR TENG (58 V and 5.8 µA) and NR-CF TENG (104 V and 9.8 µA). The electrical outputs showed an increasing trend with Ag nanoparticle concentration. However, preparing Ag nanoparticles at high concentrations led to the precipitation of Ag with a large particle size, which was not suitable for use.

**Figure 7:**
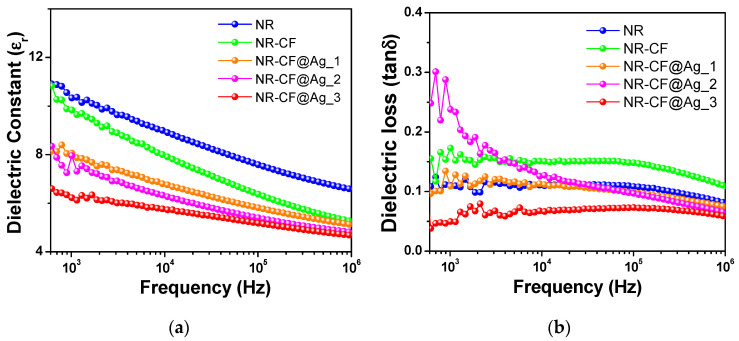
(**a**) Dielectric constants and (**b**) dielectric loss versus frequency of the NR, NR–CF, and NR–CF@Ag_1–3 composite films.

The role of Ag nanoparticle in the improvement of TENG performance was examined by measuring the dielectric properties of the NR–CF@Ag composite films to probe the charge retention properties of the films, which are presented in Figure 7. It was noted that the dielectric constant of the NR–CF@Ag was reduced with increasing Ag concentration (Figure 7a), which was lower than those of NR and NR–CF, respectively, whereas the dielectric loss did not show a correlation with the electrical outputs (Figure 7b). The result from this work showed a different trend from that in our previous study on NR–Ag TENG [14], where the dielectric constant increased with increasing Ag nanoparticle concentration, and which was affected by the nanoparticle’s capping agents. In the present work, no capping agent was used for synthesizing Ag nanoparticles; therefore, the dielectric polarization was disrupted. The addition of Ag nanoparticles without the capping agent results in the connection of the particles, forming the electrical conductive path and giving rise to the reduction of the dielectric constant [46].

The electrical outputs of a contact mode TENG are governed by triboelectric charge density (σ), as expressed by open circuit voltage (*V_oc_*) and short circuit current (*I_sc_*), which are described by the following equations [47]

(1) $$V_{oc}=\frac{\sigma x\left(t\right)}{\varepsilon_{0}}$$

(2) $$I_{sc}=\frac{S\sigma d_{0}v\left(t\right)}{{\left(d_{0}+x\left(t\right)\right)}^{2}}$$

where *x*(*t*) is the working distance, *v*(*t*) is the velocity of the moving electrode, *S* is the size of the contact area, *ε*_0_ is the permittivity of free space, and *d*_0_ is the effective thickness constant.

Triboelectric charge density is dependent on the material types, surface area, and charge capacitance (dielectric constant) of the triboelectric materials [47]. It was observed in this work that the dielectric constant was reduced with increasing Ag concentration. The surface morphologies of all the NR composite samples were flat, without a change in surface morphology, implying that the size of the surface area was not significantly different among the samples. The enhancement of TENG performance was then deduced from the electrification effect due to the intrinsic property of Ag–cellulose filled NR. This suggested that the main contribution of Ag nanoparticles to the improved electrical output was the promotion of the electron transfer in the electrification event; in other words, the conductive Ag nanoparticles facilitate the electron donating ability of CF in the NR composite. Our results show a similar trend to that noted in the previous study on the cellulose-Ag, where the presence of Ag nanoparticles in cellulose was found to enhance catalytic performance toward the reduction of 4-nitrophenol (4-NP) to 4-aminophenol (4-AP) [48].

**Figure 8:**
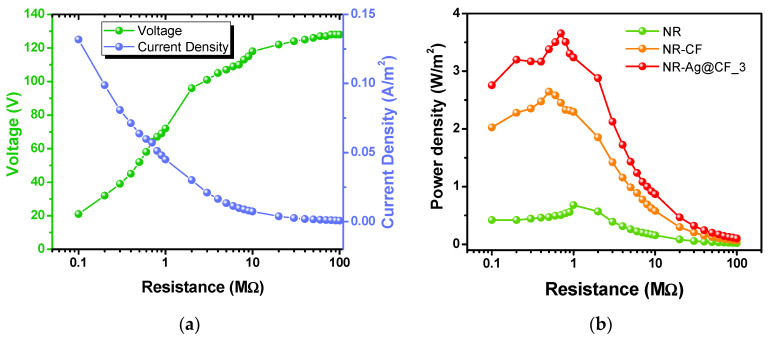

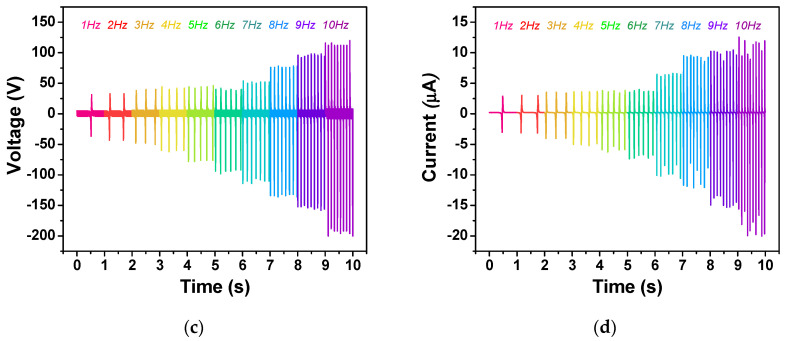
(**a**) Output voltage and current measured with the connected load resistances and (**b**) the corresponding power density of the NR–CF@Ag_3 TENG. The impact frequency dependence of (**c**) the TENG output voltage and (**d**) current outputs.

The electrical outputs of the NR–CF@Ag_3 TENG, with the connected load resistances ranging from 0.01–100 MΩ, were measured to determine the maximum delivered electrical power of the TENG. The measured output voltage and current at an impact force of 5 N and a frequency of 5 Hz are plotted against load resistances, as presented in Figure 8a. Typically, electrical outputs varied with the load resistance. Output voltage was increased and saturated at high load resistance, whereas output current showed the opposite trend. It is logical that the maximum power transfer is achieved when the internal resistance of the power source is equal to the resistance of load. The calculated power densities (*P = I*^2^*R*) [49] of the NR–CF@Ag_3 TENG compared to NR–CF and plain NR TENGs are presented in Figure 8b. The maximum power density of 3.65 W/m^2^ was achieved from the NR–CF@Ag_3 TENG at a matched load resistance of 0.7 MΩ, which was five times higher than that of the NR TENG (0.68 W/m^2^) and 1.35 time higher than that of the NR–CF TENG (2.65 W/m^2^), as given in Table 2. The enhancement of the TENG power output was due to the incorporation of conductive Ag nanoparticles with the combination of electron donating cellulose fibers.

The TENG performance was also tested at different impact frequency ranging from 1–10 Hz, as shown by the voltage and current outputs in Figure 8c,d, respectively. The outputs rose and reached up to 312 V and 32 µA at 10 Hz operation frequency. The increasing electrical output was ascribed to the movement speed of triboelectric layers, which was increased with increasing impact frequency, as described by Equation (2) above. The short contact cycle at high movement speed caused the retention of triboelectric charges, giving rise to the enhancement of electrical output [14,37]. At low working frequency, the reasonable electrical outputs were obtained. This suggested that the fabricated TENG could be used to harvest mechanical energy, with various frequency ranges.

**Figure 9:**
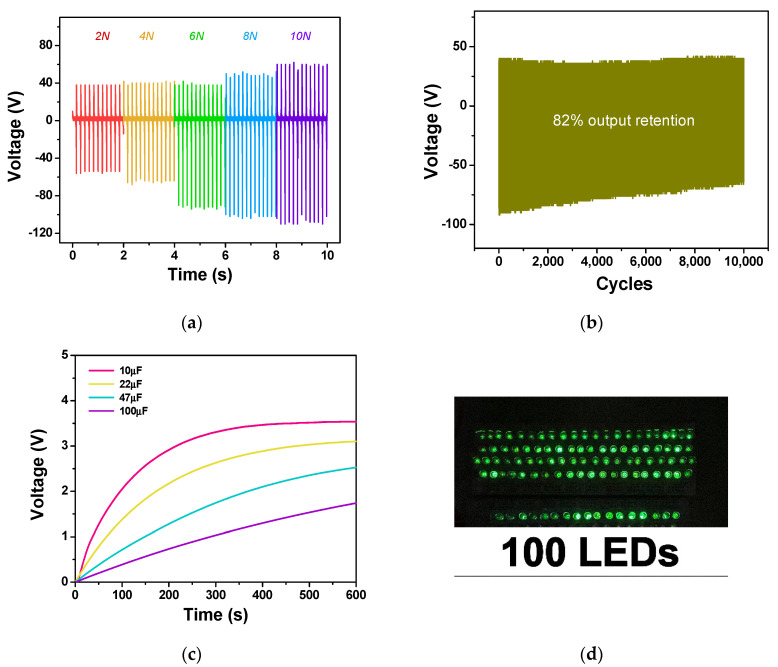
(**a**) The dependence of output voltage on the impact force; (**b**) The performance stability of TENG over 10,000 impact cycles; (**c**) voltage profile of the commercial capacitors charged by the NR–CF@Ag_3 TENG; (**d**) application of TENG to light up 100 commercial green LEDs.

The influence of the impact force on TENG performance was also investigated. The electrical outputs of the TENG tested at various impact forces from 2–10 N are displayed in Figure 9a. It was shown that the TENG outputs increased with the greater impact force. When the triboelectric layers were pressed by the applied force, their thicknesses were reduced, leading to the increased charge capacitance [14], as described by C = *ε*_0_*ε_r_A/d*, where *A* is the contact area and *d* is the thickness of the triboelectric layer. The greater the impact force, the larger the film deformation, resulting in the higher electrical output of the TENG.

The performance stability of the TENG was also tested with the constant applied force of 5 N at 5 Hz impact frequency for 10,000 cycles. It was found that the NR–CF@Ag TENG exhibited good performance stability with 82% output retention, as presented in Figure 9b. The reduction in performance stability could be due to the mechanical degradation of the NR composites. This could be explained by the fact that the NR composite was fabricated from a prevulcanized latex, meaning that it already contained a cross-linking agent. Adding CF did not change the chemical structure of the NR composites, as evidenced by the FTIR results (Figure 5b). This suggested that CF did not improve the mechanical strength of the composites, and it could disrupt the cross-linking reaction in NR due to the large fiber size [50]. Our fabricated TENG showed performance stability superior to that of the TENG made from the nanofiber/microsphere hybrid membranes (77% retention) [51] and comparable to that of our previously reported cellulose paper-based TENG (85% retention) [37] over the same number of impact cycles. The application of the fabricated TENG as a power source was also demonstrated to charge commercial capacitors by the use of a bridge rectifier. Figure 9c shows the voltage profiles of the 10, 22, 37, and 100 µF capacitors, which were charged by the NR–CF@Ag_3 TENG under the impact force of 5 N and at 5 Hz frequency. The TENG was able to charge a device as small as a 10 µF to 3.5 V in 400 s and as large as a 100 µF capacitor to 1.5 V in 500 s (~8 min). The generated electrical power from TENG was also able to instantaneously operate 100 commercial green LEDs, as displayed in Figure 9d. These results suggested the potential application of the NR–CF@Ag TENG as a power source for microelectronic devices.

## 4. Conclusions

The high performance TENG was fabricated from natural based materials. NR–CF composite film, with the incorporation of Ag nanoparticles, was found to improve the energy conversion performance of the NR-based TENG. The electron donation property of cellulose, as well as the free electron-rich Ag nanoparticles, are responsible for promoting the electrification effect. With the optimized Ag content in the NR-CF composite, the maximum power density of 3.65 W/m^2^ was obtained, which was five times higher than that of the unmodified NR TENG. The fabricated TENG was able to generated electricity which could be used as a power source for microelectronic devices. This work has proposed an effective approach to enhance the natural-based TENG toward the development of a green and sustainable power source.

## Figures and Tables

**Table 1:** Electrical output voltage (*V_pp_*) and current (*I_pp_*) of NR, NR–CF, and NR–CF@Ag_1–3 TENGs.

Specimens	*V_pp_* (V)	*I_pp_* (µA)
NR	58	5.8
NR–CF	104	9.8
NR–CF@Ag_1	112	10.2
NR–CF@Ag_2	120	11.8
NR–CF@Ag_3	128	12.4

**Table 2:** Power density of the NR–CF@Ag_3 TENG compared to the NR and NR–CF TENGs.

TENGs	Power Density (W/m^2^)
NR–CF@Ag_3	3.65
NR–CF	2.65
NR	0.68

## Data Availability

The data presented in this study are available on request from the corresponding author.
